# Outcomes of COVID-19 in a cohort of pediatric patients with rheumatic diseases

**DOI:** 10.1186/s12969-021-00568-4

**Published:** 2021-06-21

**Authors:** D. Sofia Villacis-Nunez, Christina A. Rostad, Kelly Rouster-Stevens, Arezou Khosroshahi, Shanmuganathan Chandrakasan, Sampath Prahalad

**Affiliations:** 1 Department of Pediatrics, Division of Pediatric Rheumatology, Emory University School of Medicine, Atlanta, USA; 2 Children’s Healthcare of Atlanta, Atlanta, USA; 3 Department of Pediatrics, Division of Pediatric Infectious Diseases, Emory University School of Medicine, Atlanta, USA; 4 Department of Medicine, Division of Rheumatology, Emory University School of Medicine, Atlanta, USA; 5 Department of Pediatrics, Division of Hematology, Oncology and BMT, Emory University School of Medicine, Atlanta, USA; 6 Department of Human Genetics, Emory University School of Medicine, Atlanta, USA

**Keywords:** Rheumatic diseases, COVID-19, Pediatric rheumatology

## Abstract

**Background:**

There are few reports of COVID-19 in pediatric patients with rheumatic diseases. This study describes the clinical presentation and outcomes of COVID-19 in this population.

**Methods:**

We analyzed a single-center case series of pediatric patients with rheumatic diseases and laboratory-confirmed COVID-19. Demographic, baseline and COVID-19 associated clinical features were compared between ambulatory and hospitalized patients using univariate analysis.

**Results:**

Fifty-five cases were identified: 45 (81.8%) in the ambulatory group and 10 (18.2%) hospitalized. African American race (OR 7.78; 95% CI [1.46–55.38]; *p* = 0.006) and cardiovascular disease (OR 19.40; 95% CI 2.45–254.14; *p* = 0.001) predominated in hospitalized patients. Active rheumatic disease (OR 11.83; 95% CI 1.43–558.37; *p* = 0.01), medium/high-dose corticosteroid use (OR 14.12; 95% CI [2.31–106.04]; *p* = 0.001), mycophenolate use (OR 8.84; 95% CI [1.64–63.88]; *p* = 0.004), rituximab use (OR 19.40; 95% CI [2.45–254.14]; p = 0.001) and severe immunosuppression (OR 34.80; 95% CI [3.94–1704.26]; p = < 0.001) were associated with increased odds of hospitalization. Fever (OR 7.78; 95% CI [1.46–55.38]; *p* = 0.006), dyspnea (OR 26.28; 95% CI [2.17–1459.25]; *p* = 0.003), chest pain (OR 13.20; 95% CI [1.53–175.79]; *p* = 0.007), and rash (OR 26.28; 95% CI [2.17–1459.25]; p = 0.003) were more commonly observed in hospitalized patients. Rheumatic disease flares were almost exclusive to hospitalized patients (OR 55.95; 95% CI [5.16–3023.74]; *p* < 0.001).. One patient did not survive.

**Conclusions:**

Medium/high-dose corticosteroid, mycophenolate and rituximab use, and severe immunosuppression were risk factors for hospitalization. Fever, dyspnea, chest pain, and rash were high-risk symptoms for hospitalization. Rheumatic disease activity and flare could contribute to the need for hospitalization.

## Background

COVID-19 results from infection with SARS-CoV-2, a novel coronavirus, and has been reported to have a milder disease course in children compared to adults [1, 2]. However, the known susceptibility to infections in patients with rheumatic diseases has been of great concern for pediatric rheumatologists during the pandemic [3, 4]. Outcomes from the largest case series of adults with rheumatic diseases reported 91% survival, despite a 50% hospitalization rate [5]. Although some studies of COVID-19 in rheumatic disease patients include a few pediatric cases, with overall favorable outcomes, pediatric data remain scarce [6–9]. The aim of our study was to analyze the clinical presentation and outcomes of COVID-19 in a case series of pediatric patients with rheumatic diseases, and to identify risk factors associated with adverse outcomes.

## Methods

Pediatric patients (0–21 years) with rheumatic diseases followed at Children’s Healthcare of Atlanta (CHOA) who had laboratory-confirmed SARS-CoV-2 infection from May 2020 to January 2021 were retrospectively identified. Diagnosis was established either through patient/family report of positive SARS-CoV-2 test (serology or polymerase chain reaction (PCR)) or by positive test performed at CHOA. The present study was approved by the Institutional Review Board (IRB) at CHOA via waiver of informed consent (IRB number STUDY00000771).

Cases were classified into two groups based on need for hospitalization. Demographic (age, sex, race/ethnicity), and clinical data (baseline rheumatic disease diagnosis/activity, comorbidities, medications, degree of immunosuppression, COVID-19 exposure/symptoms, immunosuppression management, and outcomes) were collected through chart review.

Rheumatic disease activity was assessed using the Systemic Lupus Erythematosus Disease Activity Index-2000 (SLEDAI-2 K) and Juvenile Arthritis Disease Activity Score (JADAS) when indicated. If unable to calculate, physician assessment of disease activity was utilized. Active disease was defined as a SLEDAI-2 K score ≥ 6, JADAS-10 score > 2, or rheumatologist assessment indicating active disease.

The level of immunosuppression was defined as severe if the subject reported use of one or more of: intravenous (IV) cyclophosphamide (within 3 months), rituximab (within 6 months), medium/high-dose (≥10 mg/day) oral corticosteroids (CS), outpatient IV CS. Patients on immunomodulators not meeting these criteria were considered mild to moderately immunosuppressed.

Demographic and clinical features were compared between groups using Fisher exact test (with corresponding Odds ratios (OR) and 95% confidence intervals (CI)) for categorical variables, and Mann-U-Whitney test for continuous variables, employing R software. *P* values ≤0.05 were considered statistically significant.

## Results

### Patient cohort, demographics, and baseline clinical features

Fifty-five cases were identified: 10 (18.2%) required hospitalization and 45 (81.8%) received ambulatory care. Demographics and baseline clinical features are outlined in Table 1. Age was similar between groups. The racial and ethnic distribution differed between groups: a higher proportion of African American patients were encountered in the hospitalized group (OR 7.78, 95% CI [1.46–55.38]; *p* = 0.006), while Caucasian patients predominated in the ambulatory group (OR 0.14, 95% CI [0.01–0.83]; *p* = 0.01).

**Table 1:** Demographic and baseline clinical features of children with rheumatic diseases and COVID-19

Category	Total ***n*** = 55		Hospitalized n = 10		Ambulatory ***n*** = 45		OR (95% CI)	P
**Demographics**								
Age in years	16	(14–18)	17	(16–19)	16	(13–17)		0.07
Female	43	(78.2)	7	(70.0)	36	(80.0)	0.59 (0.11–4.22)	0.67
African American	17	(30.9)	7	(70.0)	10	(22.2)	7.78 (1.46–55.38)	0.006
Asian	1	(1.8)	0	(0)	1	(2.2)	0 (0–175.02)	1
Caucasian	31	(56.4)	2	(20.0)	29	(64.4)	0.14 (0.01–0.83)	0.01
Hispanic or Latino	6	(10.9)	1	(10.0)	5	(11.1)	0.89 (0.02–9.54)	1
**Baseline clinical features**								
Comorbidities	27	(49.1)	6	(60.0)	21	(46.7)	1.69 (0.35–9.35)	0.50
*Primary rheumatic disease*								
Juvenile Idiopathic arthritis	17	(30.9)	1	(10.0)	16	(35.6)	0.21 (0.004–1.73)	0.15
Systemic lupus erythematosus	14	(25.5)	5	(50.0)	9	(20.0)	3.88 (0.72–21.23)	0.1
Juvenile Dermatomyositis	6	(10.9)	0	(0)	6	(13.3)	0 (0–3.94)	0.58
Other	18	(32.7)	4	(40.0)	14	(31.1)	1.46 (0.26–7.38)	0.71
Active rheumatic disease	28	(50.9)	9	(90.0)	19	(42.2)	11.83 (1.43–558.37)	0.01
*Immunomodulatory medications*								
Oral corticosteroids < 10 mg	8	(14.5)	3	(30.0)	5	(11.1)	3.33 (0.42–22.33)	0.15
Oral corticosteroids ≥10 mg	10	(18.2)	6	(60.0)	4	(8.9)	14.12 (2.31–106.04)	0.001
IV pulse corticosteroids	3	(5.5)	0	(0.0)	3	(6.7)	0 (0–11.40)	1
Hydroxychloroquine	27	(49.1)	7	(70.0)	20	(44.4)	2.89 (0.56–19.35)	0.18
Other cDMARDs^a^	20	(36.4)	2	(20.0)	18	(40.0)	0.38 (0.03–2.22)	0.3
Mycophenolate	16	(29.1)	7	(70.0)	9	(20.0)	8.84 (1.64–63.88)	0.004
Tofacitinib	2	(3.6)	1	(10.0)	1	(2.2)	4.69 (0.06–390.81)	0.33
Intravenous immunoglobulin	4	(7.3)	0	(0.0)	4	(8.9)	0 (0–7.15)	1
Cyclophosphamide	4	(7.3)	1	(10.0)	3	(6.7)	1.54 (0.03–21.94)	0.56
TNFi	14	(25.5)	0	(0.0)	14	(31.1)	0 (0–1.17)	0.05
Rituximab	7	(12.7)	5	(50.0)	2	(2.2)	19.40 (2.45–254.14)	0.001
Other bDMARDs^b^	8	(14.5)	2	(20.0)	6	(13.3)	0.73 (0.01–7.29)	1
None	3	(5.5)	0	(0.0)	3	(6.7)	0 (0–11.40)	1
Severe Immunosupression^c^	17	(32.7)	9	(90.0)	8	(19.0)	34.80 (3.94–1704.26)	< 0.001

Numerical variables are expressed as median (IQR), and categorical variables as n (%)

Abbreviations: *CI* Confidence Interval; *bDMARDs* biologic disease modifying anti-rheumatic drugs; cDMARDs: conventional disease modifying anti-rheumatic drugs; OR: Odds ratio; IQR: Interquartile range; TNFi: Tumor necrosis factor alpha inhibitors. ^a^Methotrexate (*n* = 12), leflunomide (n = 5), sulfasalazine (n = 3). ^b^Abatacept (n = 3), tocilizumab (n = 2), ustekinumab (*n* = 1), anakinra (n = 1), belimumab (n = 1). ^c^*n* = 52: Patients off immunomodulators excluded

The frequency of underlying comorbidities was comparable between groups. Reported comorbidities consisted of: asthma (*n* = 10), hypertension (*n* = 7), obesity (*n* = 5), inflammatory bowel disease (*n* = 4), anemia of chronic disease (*n* = 2), immunoglobulin G deficiency (n = 2), hypothyroidism (n = 2), end-stage renal disease on chronic dialysis (n = 2), and one patient each had pulmonary hypertension, chronic kidney disease, post-renal transplant status, left ventricular hypertrophy, chronic diarrhea, cholestasis, adrenal insufficiency, hyperparathyroidism, thyroid disease, chronic neutropenia, sickle cell disease, recurrent infections, chronic deep venous thrombosis, and Wilms tumor. No smoking or diabetes were identified in either group. Patients with cardiovascular disease, namely left ventricular hypertrophy and hypertension, were more likely to be in the hospitalized group (OR 19.40, 95% CI [2.45–254.14]; *p* = 0.001). No significant differences were observed regarding the presence of the remaining specific comorbidities between groups.

Juvenile idiopathic arthritis (JIA), systemic lupus erythematosus (SLE), and juvenile dermatomyositis (JDM) were the most common primary rheumatic diagnoses among children with COVID-19 (Table 1). Undifferentiated connective tissue disease (*n* = 3), idiopathic uveitis (n = 3), Behcet’s disease (n = 3), overlap syndrome (*n* = 2), sarcoidosis (n = 2), rheumatoid arthritis (n = 2), systemic scleroderma (*n* = 1), Takayasu’s arteritis (n = 1), and Anti-neutrophilic cytoplasmic antibody (ANCA)-associated vasculitis (n = 1) were also identified as primary rheumatic diagnoses. Five patients had an additional rheumatic comorbidity: SLE+ history of macrophage activation syndrome (n = 2), SLE+ rheumatoid arthritis (n = 1), JIA+ Sjogren’s syndrome (n = 1), ANCA-associated vasculitis+ anti-glomerular basement membrane disease. Active disease was more commonly encountered in the hospitalized group (OR 11.83, 95% CI [1.43–558.37]; *p* = 0.01).

The majority of patients in both groups were taking at least one immunomodulatory medication, as detailed in Table 1. Besides those, additional immunosuppressants used in the ambulatory group included apremilast (*n* = 1), azathioprine (n = 1), cyclosporine (n = 1), tacrolimus (n = 1), and colchicine (n = 1). NSAID use was observed in 5 (50%) patients in the hospitalized group and 21 (46.7%) patients in the ambulatory group (*p* = 1). The use of medium/high-dose CS (OR 14.12; 95% CI [2.31–106.04]; *p* = 0.001), mycophenolate (OR 8.84; 95% CI [1.64–63.88]; *p* = 0.004) and rituximab (OR 19.40; 95% CI [2.45–254.14]; p = 0.001) increased the odds of hospitalization. Patients using tumor necrosis factor-alpha inhibitors (TNFi) had decreased odds of admission (OR 0; 95% CI [0–1.17]; *p* = 0.05). No patients receiving intravenous immunoglobulin (IVIG) or IV pulse CS were hospitalized. No significant differences regarding use of other DMARDs were observed. Severely immunosuppressed individuals had increased odds of hospitalization (OR 34.80; 95% CI [3.94–1704.26]; *p* < 0.001).

### COVID-19 exposure, symptoms and diagnosis

Exposure to a close contact with confirmed COVID-19 was reported by 5 (50%) patients in the hospitalized group versus 26 (57.8%) patients in the ambulatory group (*p* = 0.75).

Table 2 lists patient reported symptoms for both groups. Patients with fever, dyspnea, chest pain, rash and myalgias had increased odds of hospitalization (*p* < 0.05 for each comparison).

**Table 2:** Clinical presentation of COVID-19 in children with rheumatic diseases

Symptoms	Total *n* = 55		Hospitalized *n* = 10		Ambulatory *n* = 45		OR (95% CI)	*P*
Fever	17	(30.9)	7	(70.0)	10	(22.2)	7.78 (1.46–55.38)	0.006
Rhinorrhea/Congestion	13	(23.6)	2	(20.0)	11	(24.4)	0.78 (0.07–4.79)	1
Cough	20	(36.4)	4	(40.0)	16	(35.6)	1.20 (0.22–5.99)	1
Dyspnea	5	(9.1)	4	(40.0)	1	(2.2)	26.28 (2.17–1459.25)	0.003
Chest pain	6	(6)	4	(40.0)	2	(4.4)	13.20 (1.53–175.79)	0.007
Myalgias	12	(21.8)	5	(50.0)	7	(15.6)	5.21 (0.94–30.12)	0.03
Abdominal pain	4	(7.3)	2	(20.0)	2	(4.4)	5.14 (0.32–80.65)	0.15
Anorexia/Nausea/Emesis	8	(14.5)	3	(30.0)	5	(11.1)	3.33 (0.42–22.34)	0.15
Diarrhea	10	(18.2)	4	(40.0)	6	(13.3)	4.18 (0.67–24.99)	0.07
Anosmia/Ageusia	14	(25.5)	2	(20.0)	12	(26.7)	0.69 (0.06–4.21)	1
Rash	5	(9.1)	4	(40.0)	1	(2.2)	26.28 (2.17–1459.25)	0.003
Fatigue/Malaise	16	(29.1)	2	(20.0)	14	(31.1)	0.56 (0.05–3.34)	0.7
Sore throat	12	(21.8)	2	(20.0)	10	(22.2)	0.88 (0.08–5.50)	1
Headache	12	(21.8)	2	(20.0)	10	(22.2)	0.88 (0.08–5.50)	1
Asymptomatic	10	(18.2)	0	(0.0)	10	(22.2)	0 (0–1.92)	0.18

Numerical variables are expressed as median (IQR), and categorical variables as n (%)

Abbreviations: *CI* Confidence Interval; Odds ratio: *OR*; *IQR* Interquartile range

Ten patients (18.2%) were asymptomatic, all in the ambulatory group. Of those, one patient had a lung infiltrate in a surveillance chest tomography for metastatic Wilms tumor, prompting SARS-CoV-2 testing. The remaining 9 patients were tested due to exposure to a close contact with confirmed COVID-19 or during routine screening at their educational institution.

Fifteen patients had confirmatory PCR testing performed at CHOA; the rest were diagnosed at an outside facility. Of the 10 hospitalized patients, three were known to be SARS-CoV-2 positive prior to admission: patients 1 and 9 were tested one week earlier, with subsequent worsening clinical status requiring hospitalization; for patient 3, PCR test results became available while still in the emergency room.

COVID-19 diagnosis was reported to Rheumatology during the acute infection period in 28 (62.2%) ambulatory and 9 (90%) hospitalized patients; the report was delayed by up 3 months for 17 (37.8%) ambulatory patients, and by 6 weeks for 1 (10%) patient admitted to an outside hospital.

### Complications, management and outcomes

Immunomodulatory management during the acute COVID-19 period did not differ between groups: medications were temporarily discontinued in 5 (50%) hospitalized patients and 16 (38.1%) ambulatory patients on immunosuppression (*p* = 0.50).

Rheumatic disease flares were observed in 6 hospitalized patients versus 1 (2.2%) ambulatory patient (OR 55.95; 95% CI [5.16–3023.74]; *p* < 0.001). Disease flare was preceded by medication discontinuation in two patients (7 and 33). Patient 33 experienced gastrointestinal symptoms (without fever or other organ involvement) from Behcet’s disease after holding adalimumab for 2 weeks; this was successfully managed by restarting the medication. Patient 7 developed flare symptoms after running out of anakinra; this improved after anakinra re-introduction and increased CS doses.

In the ambulatory group, COVID-19 treatment was largely symptomatic. Inpatient care, complications and outcomes of hospitalized patients are detailed in Table 3. Four patients required Intensive Care Unit (ICU) admission. One patient died, after having required mechanical ventilation and extracorporeal membrane oxygenation. The possibility of multisystem inflammatory syndrome in children (MIS-C) was considered but ultimately ruled out in patients 7, 9 and 10 (Table 3), based on lack of cardiac involvement, gastrointestinal symptoms explained by the presence of co-infection, and overall evolution of symptoms. No confirmed MIS-C cases were identified in this cohort. No additional late complications were encountered after a median post-COVID-19 follow-up period of 48 days.

**Table 3:** Clinical features and hospital course of children with rheumatic diseases hospitalized with COVID-19

Patient No.	Baseline features					Immuno-suppression management	Symptoms	Inpatient care and therapies								Rheumatic disease flare diagnosis^c^	Flare management	Other complications	Final outcome
	Age^a^/Sex	Rheumatic disease diagnosis	Active disease	Comorbidities	Immuno-suppression regimen			ICU LOS^b^	Total LOS^b^	No. of hospitalizations	Remdesivir	Convalescent plasma	Respiratory support	IV antibiotics	Anticoagulation				
1	16/F	SLE (with ILD)	N		HCQ, PDN 2.5 mg daily, MMF, RTX	Continued	Fever, cough, dyspnea	0	2	1	N	N	N	Y	N	None		Pneumonia	Survived
2	19/F	SLE (with LN)	Y	HTN	HCQ, PDN 40 mg daily	Continued	Fever, cough, dyspnea, anosmia/ageusia	1	5	2	N	N	LFNC	Y	Y	Before	IVMP	Pneumonia, AKI	Survived
3	17/F	Overlap sd. (with ILD)	Y		HCQ, PDN 30 mg daily, MMF, IV CYC	MMF and HCQ held	Fever, sore throat, dyspnea, myalgia, diarrhea, vomiting	4	8	1	Y	Y	HFNC	Y	Y	Before	Increased PDN dose		Survived
4	19/F	SLE+ RA	Y		HCQ, PDN 10 mg daily, MTX, MMF, RTX	Continued	Myalgia, chest pain	0	4	1	N	N	N	N	N	Before	IVMP, increased PDN dose		Survived
5	19/F	JIA	Y		Tofacitinib	Continued	Fever, abdominal pain	0	2	1	N	N	N	Y	N	None		Urosepsis	Survived
6	19/F	SLE (with LN)	Y	Hyperparathyroidism, LVH, HTN, anemia of chronic disease, s/p renal transplant	HCQ, PDN 5 mg daily, tacrolimus, MMF, RTX	MMF held	Fever, cough, diarrhea, anosmia/ageusia, rhinorrhea, fatigue, chest pain, rash	4	17	1	N	Y	HFNC	Y	Y	Concurrent	IVMP, increased PDN dose, IV CYC	Renal failure requiring HD, pneumonia	Survived
7	17/M	SLE+ MAS	Y	HTN	HCQ, PDN 20 mg daily, MMF, ANK	ANK held^d^	Fever, myalgias, headaches, vomiting, diarrhea, rash ^f^	0	4	1	N	N	N	Y	N	After	IVMP, increased PDN dose	C. difficile colitis	Survived
8	16/M	Sarcoidosis (with ILD)	Y	SCD, Cholestasis, pulmonary HTN	PDN 30 mg daily, MMF	MMF held	Myalgia	0	5	1	Y	N	LFNC	N	Y	None			Survived
9	14/M	Overlap sd. (with LN)	Y	Asthma, HTN	HCQ, PDN 60 mg daily, MMF RTX	Continued	Rash, chest pain	0	3	1	N	N	N	N	Y	None		Conjunctivitis 3 weeks post-admission^e^	Survived
10	14/F	AAV+ anti-GBM disease (with RLD)	Y	ESRD on PD, HTN, acquired IgG deficiency	PDN 5 mg daily, leflunomide, RTX	Leflunomide held	Fever, fatigue, sore throat, nausea/vomiting, diarrhea, abdominal pain, dyspnea, chest pain, rash, myalgias ^f^	26	41	3	N	N	MV/ ECMO	Y	N	After	PLEX, IVMP, IVIG, IV CYC, RTX	Pneumonia, C. difficile colitis, cytokine storm requiring ANK and TCZ	Deceased

*Abbreviations*: *AKI* Acute kidney injury, *ANK* Anakinra, *AAV* Anti-neutrophil cytoplasmic antibody-associated vasculitis, *BAL* Bronchoalveolar lavage, *C.* Clostridium, *CYC* Cyclophosphamide, *ECMO* Extracorporeal membrane oxygenation, *ESRD* End-stage renal disease, *F* Female, *GBM* Glomerular basement membrane, *HCQ* Hydroxychloroquine, *HD* Hemodialysis, *HFNC* High-flow nasal cannula, *HTN* Hypertension, *ICU* Intensive Care Unit, *IgG* Immunoglobulin G, *ILD* Interstitial lung disease, *JIA* Juvenile Idiopathic arthritis, *IV* Intravenous, *IVIG* Intravenous Immunoglobulin, *IVMP* Intravenous methylprednisolone pulses, *LFNC* Low-flow nasal cannula, *LOS* Length of stay, *LN* Lupus nephritis, *LVH* Left ventricular hypertrophy, *M* Male, *MAS* Macrophage activation syndrome, *MMF* Mycophenolate, *MV* Mechanical ventilation, *N* No, *No.* Number, *PCR* Polymerase chain reaction, *PD* Peritoneal dialysis, *PDN* Prednisone, *PLEX* Plasmapheresis, *RA* Rheumatoid arthritis, *RLD* Restrictive lung disease, *RTX* Rituximab, *SARS-CoV-2* Severe acute respiratory syndrome coronavirus 2, *SCD* Sickle cell disease, *Sd.* Syndrome, *SLE* Systemic lupus erythematosus, *TCZ* Tocilizumab, *Y* Yes

^a^In years

^b^In days

^c^in relation to acute COVID-19 diagnosis

^d^Patient ran out of anakinra 2 days prior to admission; re-started upon hospitalization

^e^No cardiac involvement or fever

^f^No cardiac involvement

## Discussion

This study describes the presentation and outcomes of a cohort of pediatric patients with rheumatic diseases and laboratory-confirmed COVID-19. Overall, approximately 18% of our patients required hospitalization and 7.3% required ICU care. Although these rates are slightly higher than those reported in the general pediatric population, our study had a limited sample size relying mostly on voluntary reporting, and laboratory confirmation was required [10, 11]. As such, some milder cases may have gone unreported, and untested and/or asymptomatic cases were likely excluded. Additionally, severe COVID-19 has been linked African American race, which was overrepresented in the hospitalized group [2, 12]. Cardiovascular disease, a known a risk factor for hospitalization in adults, was found more frequently in the hospitalized group in our cohort, potentially having influenced these patients’ outcomes [5]. Reassuringly, the vast majority of our patients did not require mechanical ventilation and survived, supporting previous reports that patients with rheumatic diseases and COVID-19 overall recover well [6–9].

Symptoms of acute COVID-19 were similar to those described in the general pediatric population [2, 10, 11]. Symptom reporting in the hospitalized group may have been less subject to recall bias than the ambulatory group. Notably, while respiratory and gastrointestinal symptoms are typically attributable to acute infection, fever, rash and myalgias are also features of rheumatic disease flares. Fever, chest pain, myalgias, rash and dyspnea predominated in hospitalized patients; furthermore, patients with active rheumatic disease were more likely to be in the hospitalized group. These observations suggests that the interplay between rheumatic disease activity and flare, and severe COVID-19 may influence the need for admission, as proposed by Ye et al. [6]

Interestingly, while all JDM patients were managed in the ambulatory setting, SLE diagnosis was more common in the hospitalized group, although this association did not reach statistical significance. This raises the possibility of disease type playing a role in the outcomes as well. Indeed, the pattern of immune activation in patients with severe COVID-19 has been recently described, and is reported to bear many similarities to active SLE [13]. Moreover, therapy and presence of comorbidities vary by disease, offering another possible explanation for this observation.

Echoing the findings of Gianfrancesco et al., medium/high-dose corticosteroid use was associated with increased odds of hospitalization [5]. Similar associations were observed for mycophenolate and severe immunosuppression; the latter may have been driven by the effect of medium/high-dose steroid. Patients receiving rituximab therapy were also more likely to be in the hospitalized group. While mycophenolate use has not been reported to increase the hospitalization risk in adults, rituximab use was found to be associated with higher rates of hospitalization and death from COVID-19 in a small cohort of adult patients with rheumatic diseases [14]. Although these drugs have distinct mechanisms of action, the target cells of these medications do ultimately interact to elicit the adaptive immune response; thus, it is plausible that the chronic blunting of this response could predispose the host to a more severe COVID-19 course. We were unable to elucidate if our findings were directly related to the immunosuppressive effects of these medications versus the underlying rheumatic disease and its severity, or a combination of both. Nonetheless, these results could set the ground for larger, multicenter studies to confirm these associations.

Aligning with the previously reported inverse association of TNFi and hospitalization in adults with rheumatic diseases, and the suggested absence of severe COVID-19 in a small cohort of pediatric patients with JIA on TNFi, our TNFi recipients had decreased odds of hospitalization; in fact, no patients on TNFi were hospitalized [5, 15]. Similar observations were noted for IVIG and IV pulse CS users, a previously unreported trend that merits further exploration. Therapy with other DMARDs or NSAIDs did not influence hospitalization status, comparable to adults [5, 16].

Importantly, management of immunomodulators did not significantly differ between groups. Withholding immunosuppression in patients with rheumatic diseases and symptomatic COVID-19 has been recommended by the American College of Rheumatology (ACR) [17]. Each case should be assessed individually, as both COVID-19 and rheumatic disease exacerbations entail risks for hospitalization, especially considering that disease flare usually warrants an increase in immunosuppression.

Our study’s major strength is that, to our knowledge, this is the largest case series describing laboratory-confirmed COVID-19 in pediatric patients with rheumatic diseases. Limitations include its retrospective nature, and the method of patient selection which precludes us from calculating population-based incidence and obtaining generalizable conclusions regarding severity compared to children without rheumatic diseases. The relatively small sample size precluded us from testing for collinearity between variables and performing multivariable analyses. Despite these limitations, we were able to achieve our aim of conveying the outcomes of COVID-19 in a cohort of pediatric rheumatic disease patients, setting precedent for larger studies needed to confirm the potential associations described in this manuscript. Multicenter studies would be useful to more comprehensively define the spectrum of COVID-19 and its natural history in children with rheumatic diseases.

## Conclusions

In conclusion, the need for hospitalization for COVID-19 in this cohort of pediatric patients with rheumatic diseases was associated with African American race, cardiovascular disease, active rheumatic disease, medium/high-dose prednisone use, mycophenolate use, rituximab use and severe immunosuppression. Fever, dyspnea, chest pain, rash and myalgias were high-risk symptoms for hospitalization which should be closely monitored when present. Decisions to continue or discontinue rheumatic disease therapy should be made on a case-by-case basis, following current ACR guidelines.

## Data Availability

The datasets used and/or analyzed during the current study are available from the corresponding author on reasonable request.

## Abbreviations

ACR: American College of Rheumatology
CHOA: Children’s Healthcare of Atlanta
CI: Confidence Interval
COVID-19: Coronavirus Disease 19
CS: Corticosteroids
DMARDs: Disease modifying anti-rheumatic drugs
IRB: Institutional Review Board
ICU: Intensive Care Unit
IQR: Interquartile range
IV: Intravenous
IVIG: Intravenous Immunoglobulin
JDM: Juvenile Dermatomyositis
JIA: Juvenile Idiopathic Arthritis
JADAS: Juvenile Arthritis Disease Activity Score
MIS-C: Multisystem inflammatory syndrome in children
NSAIDs: Non-Steroidal anti-inflammatories
OR: Odds ratio
PCR: Polymerase chain reaction
SARS-CoV-2: Severe Acute Respiratory Syndrome Coronavirus 2
SLE: Systemic lupus erythematosus
SLEDAI-2 K: Systemic Lupus Erythematosus Disease Activity Index-2000
TNFi: Tumor necrosis factor alpha inhibitors
